# In vivo interaction of anti-cancer drugs with misonidazole or metronidazole: methotrexate, 5-fluorouracil and adriamycin.

**DOI:** 10.1038/bjc.1980.334

**Published:** 1980-12

**Authors:** I. F. Tannock

## Abstract

I have studied the effects on growth of two tumours in mice and on host toxicity, of combining Misonidazole (MISO) or Metronidazole (METRO) with Methotrexate (MTX)-5-fluorouracil (FU) or Adriamycin (ADR). The nitroimidazoles alone had no effect on the growth of either tumour, but MISO (1 mg/g) led to a small increase in delay to regrowth of the 16/C mammary carcinoma but not the KHT fibrosarcoma, when given after X-irradiation. MTX was active only against the KHT tumour, and growth delay was not increased by the addition of MISO or METRO. FU delayed growth of both tumours, and growth delay was increased slightly by single-dose MISO. ADR was active only against the 16/C tumour, and delay to regrowth was increased by adding MISO. Host toxicity assessed by death and loss of body weight was much greater when MISO or METRO were added to MTX, and a little greater when they were added to FU. ADR plus MISO caused no deaths and no greater loss of body weight than ADR alone. The addition of MISO to treatment with anticancer drugs led to a slightly greater and more prolonged myelosuppression. METRO and MISO increase the anti-tumour effects of some anti-cancer drugs, but may also increase host toxicity. Nitroimidazoles should be used with caution in combination with chemotherapy.


*Br. J. Cancer* (1980) **42**, 861

### *IN VIVO* INTERACTION OF ANTI-CANCER DRUGS WITH MISONIDAZOLE OR METRONIDAZOLE: METHOTREXATE, 5-FLUOROURACIL AND ADRIAMYCIN

I. F. TANNOCK

*From the Departments of Medicine and Physics, Ontario Cancer Institute and The Princess Margaret Hospital, Toronto, Canada M4X 1K9*

Received 24 June 1980 Accepted 26 August 1980

**Summary.—I have studied the effects on growth of two tumours in mice and on host toxicity, of combining Misonidazole (MISO) or Metronidazole (METRO) with Methotrexate (MTX), 5-fluorouracil (FU) or Adriamycin (ADR). The nitroimidazoles alone had no effect on the growth of either tumour, but MISO (1 mg/g) led to a small increase in delay to regrowth of the 16/C mammary carcinoma but not the KHT fibrosarcoma, when given after X-irradiation.**

**MTX was active only against the KHT tumour, and growth delay was not increased by the addition of MISO or METRO. FU delayed growth of both tumours, and growth delay was increased slightly by single-dose MISO. ADR was active only against the 16/C tumour, and delay to regrowth was increased by adding MISO.**

**Host toxicity assessed by death and loss of body weight was much greater when MISO or METRO were added to MTX, and a little greater when they were added to FU. ADR plus MISO caused no deaths and no greater loss of body weight than ADR alone. The addition of MISO to treatment with anticancer drugs led to a slightly greater and more prolonged myelosuppression.**

**METRO and MISO increase the anti-tumour effects of some anti-cancer drugs, but may also increase host toxicity. Nitroimidazoles should be used with caution in combination with chemotherapy.**

MANY solid tumours have a poor blood supply and contain hypoxic cells that are resistant to treatment with radiation. Hypoxic and other poorly nourished cells may also limit the effectiveness of chemotherapy for a number of reasons. The concentration of anti-cancer drugs in such cells may be low because of their position relative to the blood supply, as demonstrated in tumours and spheroids by studying the fluorescence of ADR (Ozols *et al.*, 1979; Sutherland *et al.*, 1979). Also, poorly nourished tumour cells tend to be slowly proliferating (Tannock, 1968, 1970; Hirst & Denekamp, 1979) whereas most anti-cancer drugs are more active against rapidly proliferating cells (Tannock, 1978). Finally, drug uptake or activity might be influenced by the nutritional state of the cells or by neighbouring tumour necrosis.

If hypoxic cells in solid tumours are resistant to some anti-cancer drugs, an improved therapeutic index (*i.e.* ratio of tumour damage to normal tissue damage) might be achieved by including in drug combinations agents with selective toxicity for hypoxic cells. MISO and METRO are drugs that have been found to have greater toxicity for hypoxic cells than for aerobic cells in tissue culture (*e.g.* Mohindra & Rauth, 1976; Moore *et al.*, 1976; Stratford & Adams, 1977; Taylor & Rauth, 1978) and to induce necrosis of hypoxic cells in the centre of multicellular spheroids (Sridhar *et al.*, 1976). At high concentration, the same drugs have good penetration into hypoxic regions of tumours, and have been shown to kill hypoxic cells in some, but not all, mouse tumours (Foster *et al.*, 1976; Brown, 1977; Denenkamp, 1978; Pederson *et al.*, 1979). Also MISO may be metabolized in hypoxic tumour regions, with release of metabolites that can kill neighbouring better-oxygenated cells (Brown, 1977; Brown & Yu, 1979).

The present series of experiments was designed to study the effect of combining MISO or METRO with conventional anti-cancer drugs on the response of two experimental tumours, and on host toxicity. Both single and multiple doses of MISO and METRO have been studied, because the half-lives of the drugs in mice ($\sim$1 h) are shorter than in man ($\sim$10 h). Methotrexate, 5-fluorouracil, and Adriamycin were chosen for study because they are used commonly to treat solid tumours in man, have selective toxicity for proliferating cells, and because ADR is known to have poor penetration from blood vessels. Experiments in which MISO or METRO were given *after* X-irradiation to the tumours are included in order to assess the *in vivo* toxicity of the drugs for hypoxic tumours.

### MATERIALS AND METHODS

*Animals and Tumours.*—C3H male mice (Flow Laboratories) at least 10 weeks old were used in all experiments. Experimental tumours were the KHT fibrosarcoma and the 16/C mammary adenocarcinoma. The KHT tumour has been serially transplanted in our laboratory, and is known to respond to some anti-cancer drugs and to contain hypoxic cells (Lin & Bruce, 1972; Hill & Bush, 1977). The 16/C tumour was obtained from the NCI tumour bank at Mason Research Laboratories, Worcester, Massachusetts, and subsequently has been serially transplanted; it is known to respond to several anti-cancer drugs including ADR and FU (Corbett *et al.*, 1978). For implantation of tumours a single cell suspension was obtained by a method described previously (Thomson & Rauth, 1974) and $\sim 2 \times 10^5$ cells were injected into the left hind leg of each animal. Both types of tumour will grow progressively from implants of 100 cells.

Growth curves for the tumours were generated as follows. Tumour diameter was recorded to the nearest 0·5 mm by passing the leg through a series of graded holes drilled in perspex, and the tumour weight was estimated from this measurement by a previously defined calibration curve. Animals were coded with numbered ear tags prior to treatment, and randomized groups of 6–8 mice received various treatments. Treatment was given when tumours had a mean diameter of 8–9 mm (weight 0·3 g). Tumour diameter and animal weight were then recorded at 1–3-day intervals by an observer who was unaware of the treatment history. Mean tumour weight and its standard error were plotted against time after treatment. Most experiments were repeated to check reproducibility.

*Drugs and Radiation.*—Methotrexate (MTX Lederle), 5-fluorouracil (FU, Roche) and Adriamycin (ADR, Adria Laboratories) were standard parenteral formulation obtained from our hospital pharmacy. Metronidazole (METRO) was donated by Poulenc (Montreal) and Misonidazole (MISO) by Roche (Welwyn Garden City, England). All drugs were diluted to an appropriate concentration with physiological saline shortly before use.

All the anti-cancer drugs were given in a fluid volume of 0·01 ml/g body weight by i.p. injection. Because of their limited solubility, MISO and METRO were injected i.p. in volumes of 0·02 or 0·05 ml/g body weight. Control animals received equal volumes of saline.

Serum concentration of MISO or METRO was measured by high-pressure liquid chromatography (HPLC, Gudaskas *et al.*, 1978; Workman *et al.*, 1978). Groups of 3 mice were killed at various times after injection of MISO or METRO, and heparin was injected shortly before death to prevent clotting. Blood samples were collected from the inferior vena cava, and the pooled blood was centrifuged. Plasma was mixed with methanol to precipitate protein, filtered and injected on to the HPLC column. Drug concentration was obtained from a previous calibration curve, derived from samples of known concentration.

Tumours in some experiments were irradiated locally using a double-headed 100kVp X-ray unit at a dose rate of 11 Gy/min (Siemann *et al.*, 1975). Mice were not anaesthetized for irradiation.

*Assessment of haematological toxicity.*—Haemoglobin levels (Hb) and white blood cell counts (WBC) were measured for individual mice. A small incision was made in the tail and 44·6 μl of blood was collected in a heparinized pipette. The blood was diluted in 10 ml saline, and analysed using a Coulter Counter

Model SSr. Blood smears were also prepared and differential counts of polymorphs and other WBCs were recorded for some samples. The method allowed serial estimation of blood counts without killing the animals.

### RESULTS

### *MISO or METRO alone*

A single i.p. injection of MISO at a dose of 1 mg/g was given in many experiments. This dose was well tolerated and usually produced only a transient weight loss, < 5%. The single-dose $LD_{50}$ for MISO is ~1·4 mg/g. Multiple i.p. injections of MISO or METRO were given in other experiments in an attempt to sustain plasma levels over 36 h. Nine doses of 0·2 mg/g (MISO) or 0·4 mg/g (METRO) were given at 4h intervals; these doses were 30–50% of $LD_{50}$ but caused no deaths, and weight loss < 5%. Any toxic deaths after higher doses of either drug occurred within 24 h and surviving mice had no apparent chronic toxicity.

Previous experiments in this laboratory have shown that peak serum levels of MISO are proportional to dose (A. M. Rauth, personal communication). Following an i.p. injection of 1 mg/g MISO, serum levels rose to about 4 mM after 15 min, and then declined rapidly with a half-life of ~1 h. Serum levels of METRO after the 1st and 9th of a series of 4h injections are shown in Fig. 1. Peak levels are similar (2 mM) so there was little accumulation of drug, and half life was longer than for MISO (1·2–1·7 h). Even after 9 injections there was little tendency to sustain a more constant drug concentration. There were marked variations in serum concentration of METRO and MISO during the course of multiple injections.

Tumour growth curves for the KHT tumour, following treatment of host animals with the multiple-dose regimens of MISO and METRO were generated in duplicate experiments, and are shown in Fig. 2. Estimates of tumour weight after treatment of either the KHT or 16/C tumours with 1 mg/kg MISO (single dose) are included in Fig. 3. MISO and METRO

FIG. 1.—Serum concentration of METRO at various times after the first (A) and ninth (B) injection of the drug in a dose of 0·4 mg/g body wt given every 4 h. Each point is obtained from HPLC analysis of pooled serum from 3 animals.

FIG. 2.—Growth curves for the KHT tumour treated with multiple doses of METRO (▲) or MISO (■) (see text) (○, control). Results are from separate experiments. Means ± s.e. for 7–8 tumours are indicated.

alone have negligible effects on tumour growth.

Haemoglobin and WBC count were measured at various times from 1–7 days after treatment with MISO (1 mg/g single dose) and compared with values for mice receiving saline. Some of these data are included in Table I. There was a tendency for Hb to fall and WBC to increase in mice that were bled serially. There were no differences in mean Hb level between mice receiving MISO or saline, but in each of 5 experiments mean WBC count was slightly lower in animals that had received MISO. However, MISO did not suppress values of WBC and polymorph counts below the range found in control animals.

*Radiation*

Tumour cells which survive moderate or large doses of radiation *in vivo* are usually hypoxic. Therefore MISO or METRO was given to tumour-bearing mice *after* 15 Gy tumour radiation to seek evidence for killing of hypoxic cells *in situ*. The drugs were given after radiation to avoid radiosensitization.

Regression and regrowth curves after irradiation of KHT and 16/C tumours are shown in Fig. 3. Single dose MISO (1 mg/g) after radiation led to a small increase in effect against the 16/C tumour but in only 1 of 2 experiments (shown in Fig. 3B) was the separation of regrowth curves significant. The same dose of MISO had no effect when given after radiation to the KHT tumour (Fig. 3A) but a higher dose (1·2 mg/g) gave short prolongation of growth delay of about 2 days (not shown).

In further experiments, the 36h course of multiple injections of MISO or METRO was commenced immediately after 15 Gy irradiation to the KHT tumour, or was given so that the radiation immediately

FIG. 3.—Growth curves for the KHT sarcoma (A) and 16/6 carcinoma (B) treated with saline (○), MISO (●) (1 mg/kg, single dose) 15 Gy X-rays (△) and 15 Gy X-rays followed immediately by MISO (▲). Means ± s.e. for 7–8 tumours are indicated.

TABLE I.—*Peripheral white blood cell count (mean ± s.e. × $10^3$) at various times after drug treatment of C3H mice*

| | Day 2 | Day 3 | Day 4 | Day 6 |
|---|---|---|---|---|
| Controls | 11·2 ± 1·1 | 9·0 ± 0·4 | 8·9 ± 0·5 | 20·6 ± 2·1 |
| Misonidazole (1 mg/g) | 8·0 ± 0·5 | 7·6 ± 0·4 | 6·6 ± 0·5 | 12·6 ± 1·7 |
| Methotrexate (75 mg/kg) | 10·8 ± 0·6 | 6·3 ± 0·6 | 11·4 ± 1·1 | 20·1 ± 1·8 |
| MTX + MISO | 7·6 ± 1·0 | 5·0 ± 0·4 | 3·6 ± 0·4 | 7·6 ± 1·1 |
| 5-Fluorouracil (60 mg/kg) | 9·5 ± 0·7 | 5·5 ± 0·7 | 8·0 ± 0·6 | 6·3 ± 1·0 |
| FU + MISO | 6·8 ± 0·3 | 6·9 ± 0·3 | 6·5 ± 0·3 | 4·3 ± 0·2 |
| Adriamycin (10 mg/kg) | 7·9 ± 0·5 | 6·1 ± 0·5 | 6·3 ± 0·6 | 12·7 ± 2·5 |
| ADR + MISO | 6·2 ± 0·6 | 6·5 ± 0·4 | 5·3 ± 0·4 | 8·5 ± 1·3 |

preceded the 5th of the 9 injections of MISO or METRO. In the latter design, radiation to the KHT tumour was given 4 h after the previous dose of MISO (0·2 mg/g) or METRO (0·4 mg/g) so that hypoxic cell sensitization is expected to be minimal. Similar results were obtained for either drug: there was a small increase in growth delay of about 2 days if radiation was delivered within a course of multiple injections of MISO or METRO, but no effect when the course of injections was begun immediately after radiation.

*Methotrexate*

MTX given as 3 injections of 25 mg/kg at 4h intervals had no effect on growth of the 16/C tumour, and the addition of MISO was also without effect. The same dose and schedule of MTX led to a delay in growth of 2–3 days for the KHT tumour, and a higher dose of 40 mg/kg/injection

FIG. 4.—Growth curves for the KHT sarcoma following treatment with saline (○), MTX (●) (40 mg/kg × 3), MTX+MISO (0·2 mg/g × 9) (□) and MTX+METRO (0·4 mg/g × 9) (△). Means ± s.e. for 6–8 tumours are indicated.

TABLE II.—*Weight loss and number of deaths in mice treated with MTX alone, or in combination with MISO or METRO*

| | % Weight loss* | Proportion of deaths† |
|---|---|---|
| MTX alone | 7·6 (5·3–10·1) | 1/47 |
| MTX+MISO (single dose) | 13·0 (9·6–16·2) | 2/19 |
| MTX+MISO (mult. dose) | 13·6 (11·5–15·7) | 3/16 |
| MTX+METRO (mult. dose) | 17·0 (16·5–17·4) | 6/16 |

* Mean (range of means in individual experiments).
† Most died 4–6 days after treatment.

given in the same schedule caused some regression and delayed tumour growth by about 4 days. The above dose schedules of MTX were combined with single dose MISO (given with the second of 3 MTX injections), or with the multiple-dose schedule of MISO or METRO (injections of MTX given with the 4th–6th injections of the nitroimidazole). Results of one of the latter experiments are shown in Fig. 4. MISO or METRO did not increase the anti-tumour effects of MTX in any experiment.

MISO and METRO added considerable toxicity when combined with MTX. There were more deaths (usually on Days 4–6 after treatment) and more weight loss in mice receiving combined treatment (Table II). Also, the fall in WBC count was lower and more prolonged when Misonidazole was added to treatment with MTX (Table I) though there was no effect on Hb level.

### 5-*Fluorouracil*

A single dose of 100 mg/kg FU delayed growth of the KHT tumour by ~3 days and of 16/C by ~5 days. The same total dose was more effective against the KHT tumour when given as a single injection than as 3 equal doses at 4h intervals. When a single dose of FU was given in the middle of a course of multiple injections of MISO or METRO to animals bearing the KHT tumour, there was no increase in growth delay over those mice receiving FU alone. A single injection of MISO (1 mg/g) given simultaneously with FU led to a small increase in growth delay of both tumours (Fig. 5).

The addition of a nitromidazole to treatment with FU increased the toxicity as measured by death and loss of body weight (Table III), though the effect was less than for MTX. The fall in WBC count was also lower and more prolonged after combined treatment than after treatment with FU alone.

TABLE III.—*Weight loss and number of deaths in mice treated with FU alone, or in combination with MISO or METRO*

| | % Weight loss* | Proportion of deaths |
|---|---|---|
| FU alone | 2·6 (0–6·2) | 0/48 |
| FU+MISO (single dose) | 7·8 (6·6–9·1) | 0/20 |
| FU+MISO (mult. dose) | 4·3 (3·6–5·0) | 2/16† |
| FU+METRO (mult. dose) | 5·6 (3·1–8·0) | 3/16† |

* Mean (range of means in individual experiments).
† 6–8 days after treatment.

FIG. 5.—Growth curves for the KHT sarcoma (A) and 16/C carcinoma (B) treated with saline (○), FU (●) (60 mg/kg in A, 100 mg/kg in B) or FU+MISO (1 mg/g) (△). Means ± s.e. for 6–7 tumours are indicated.

*Adriamycin*

Single doses of ADR up to 15 mg/kg i.p. or 20 mg/kg i.v. were ineffective against the KHT tumour, and higher doses killed the animals. In contrast, the 16/C tumour is known to be sensitive to ADR (Corbett *et al.*, 1978) and single doses of 10 or 15 mg/kg i.p. caused complete regression of some tumours (but no cures) and delay to regrowth of 11–13 days (Fig. 6). Simultaneous injection of single-dose MISO (1 mg/g) with ADR prolonged the delay to regrowth by 3–5 days (Fig. 6).

Mean loss of body weight after combined treatment with ADR and MISO (8·7%, range 4·6–12·2%) was no greater than after treatment with ADR alone (9·4%, range 6·8–14·3%). There were no treatment-related deaths in either group, and MISO could be given with 15 mg/kg i.p. of ADR (0/12 deaths) even though a slightly higher dose of ADR alone (20 mg/kg i.p.) was lethal (7/8 deaths). Myelosuppression after ADR was minimal, and although WBC count tended to be slightly lower in mice that also received MISO, the effect was not significant (Table I).

FIG. 6.—Growth curves for the 16/C carcinoma treated with saline (○), MISO (1 mg/g) (●), ADR (15 mg/kg in a), 10 mg/kg in b) (△) and ADR+MISO (▲). Means ± s.e. for 6–7 tumours are indicated.

### DISCUSSION

The present experiments were designed to provide information relevant to two related questions:

(i) Are there important interactions between anti-cancer drugs and MISO or METRO that would encourage or discourage their combination in patients?

(ii) Is there evidence for sparing of hypoxic cells by conventional chemotherapy, and for selective killing of hypoxic cells by MISO or METRO?

The first of the above questions is of current and practical importance because clinical trials of nitroimidazoles used alone or in combination with anti-cancer drugs have been proposed. Current trials of MISO as a radiation sensitizer are also including some patients receiving chemotherapy. METRO alone was inactive against colo-rectal carcinoma (Frytak *et al.*, 1978) but its role in combination chemotherapy remains to be defined. However, the results of this and the succeeding paper show that MISO and METRO may add considerable toxicity to conventional anti-cancer drugs. Introduction of such drug combinations in clinical medicine should be made with caution, and in Phase I clinical trials to study toxicity.

The data reported do not answer conclusively the second question about the importance of hypoxic cells in chemotherapy. Limited drug delivery to poorly nourished tumour cells (Ozols *et al.*, 1979), and their documented low rate of proliferation (Tannock, 1968, 1970; Hirst & Denekamp, 1979) should convey resistance to many drugs. However, there is no direct evidence that the chronically malnourished cells retain clonogenicity and the ability to re-establish the tumour. There is contrary evidence in some tumours that the hypoxic cells which convey resistance to radiation may be acutely hypoxic because of changes in blood flow (Brown 1979; Yamaura & Matsuzawa, 1979). Such transient hypoxia conveys resistance to radiation because exposure to radiation is short, but might have less influence on cell proliferation and on drug concentration, unless serum half-life were also very short. There have been few direct studies of the response to chemotherapy of hypoxic and aerobic cells in solid tumours. Cyclophosphamide was reported to spare hypoxic cells in a rat carcinoma but to have no specificity for B16 melanoma, while BCNU and nitrogen mustard were reported to spare hypoxic cells in the B16 melanoma and the KHT sarcoma respectively (Hill & Stanley, 1975; Hill & Bush, 1977; Dixon *et al.*, 1978). I am unaware of data for MTX, FU or ADR.

If chronically hypoxic cells in solid tumours are both clonogenic and resistant to conventional chemotherapy, it remains uncertain whether MISO or METRO can be given in adequate concentration to kill surviving hypoxic cells. Experiments in which these drugs have been given after radiation have demonstrated hypoxic cell toxicity in about half the reported studies (Denekamp, 1978) and in current experiments MISO at 1 mg/g increased the anti-tumour effect when given after irradiation to the 16/C but not to the KHT tumour. *In vitro* studies of the toxicity of MISO and METRO for hypoxic cells suggest that drug–cell contact time may be more important than peak concentration (Hall *et al.*, 1978; A. M. Rauth, personal communication). Sustained but lower serum concentration of the drugs is more easily achieved in man where the serum half-lives of the drugs are much longer than in mice. Multiple injections were used in current experiments to try to increase drug–cell contact time, but also had little or no effect in prolonging growth delay of the irradiated KHT tumour. Other factors that might limit the contact time between drug and hypoxic cells include a relatively short life of even "chronically hypoxic" cells in murine tumours (Tannock, 1968) and rapid reoxygenation after radiation or drugs (Hill & Bush, 1977). These factors, together with a lower temperature in peripheral murine tumours, and hence lower sensitivity of hypoxic cells to MISO or METRO, could lead to a lower probability of detecting specific toxicity for hypoxic cells in mice than in man (Stratford & Adams, 1978).

In summary, my results show that MISO may increase the effectiveness of FU and ADR against some experimental tumours, though the observed effects are small and the mechanism uncertain. Toxicity was increased when MISO or METRO were combined with MTX or FU, and the combination with MTX led to a decrease in Therapeutic Index. Increased toxicity was not demonstrated when MISO was added to ADR but further experiments to assess a range of normal-tissue toxicity would be essential before concluding that this combination might convey therapeutic benefit.

I wish to thank Mrs P. Guttman for her expert assistance and Dr R. P. Hill for his helpful suggestions. Supported by a Research Grant from the National Cancer Institue of Canada.

### REFERENCES

BROWN, J. M. (1977) Cytotoxic effects of the hypoxic cell radiosensitizer Ro-07-0582 to tumor cells *in vivo*. *Radiat. Res.*, **72**, 469.

BROWN, J. M. (1979) Evidence for acutely hypoxic cells in mouse tumours, and a possible mechanism of reoxygenation. *Br. J. Radiol.*, **52**, 650.

BROWN, J. M. & YU, N. Y. (1979) Cytotoxicity of misonidazole *in vivo* under conditions of prolonged contact of drug with the tumour cells. *Br. J. Radiol.*, **52**, 893.

CORBETT, T. H., GRISWOLD, D. P., JR, ROBERTS, B. J., PECKHAM, J. C. & SCHABEL, F. M., JR. (1978) Biology and therapeutic response of a mouse mammary adenocarcinoma (16/C) and its potential as a model for surgical adjuvant chemotherapy. *Cancer Treat. Rep.*, **62**, 1471.

DENEKAMP, J. (1978) Cytotoxicity and radiosensitization in mouse and man. *Br. J. Radiol.*, **51**, 636.

DIXON, B., MOORE, J. V. & SPEAKMAN, H. (1978) Radiobiological hypoxia of a transplanted rat tumour and the effect of treatment with cyclophosphamide. *Eur. J. Cancer*, **14**, 1383.

FOSTER, J. L., CONROY, P. J., SEARLE, A. J. & WILLSON, R. L. (1976) Metronidazole (Flagyl): Characterization as a cytotoxic drug specific for hypoxic tumour cells. *Br. J. Cancer*, **33**, 485.

FRYTAK, S., MOERTEL, C. G., CHILDS, D. S., SCHUTT, A. J. & ALBERS, J. W. (1978) Phase II study of metronidazole therapy for advanced colorectal carcinoma. *Cancer Treat. Rep.*, **62**, 483.

GUDASKAS, G. A., PALCIC, B. & KONG, S. (1978) The determination of misonidazole, metronidazole and Ro-05-9963; a class of radiosentizing agents by HPLC in plasma and urine. *Clin. Biochem.*, Suppl. II, 8.

HALL, E. J., MILLER, R., ASTOR, M. & RINI, F. (1978)

The nitroimidazoles as radiosensitizers and cytotoxic agents. *Br. J. Cancer*, **37**, 120.

HILL, R. P. & BUSH, R. S. (1977) A new method of determining the fraction of hypoxic cells in a transplantable murine sarcoma. *Radiat. Res.*, **70**, 141.

HILL, R. P. & STANLEY, J. A. (1975) The response of hypoxic B16 melanoma cells to *in vivo* treatment with chemotherapeutic agents. *Cancer Res.*, **35**, 1147.

HIRST, D. G. & DENEKAMP, J. (1979) Tumour cell proliferation in relation to the vasculature. *Cell Tissue Kinet.*, **12**, 31.

LIN, H. & BRUCE, W. R. (1972) Chemotherapy of the transplanted KHT fibrosarcoma in mice. *Ser. Haematol.*, **5**, 89.

MOHINDRA, J. K. & RAUTH, A. M. (1976) Increased cell killing by metronidazole and nitrofurazone of hypoxic compared to aerobic mammalian cells. *Cancer Res.*, **36**, 930.

MOORE, B. A., PALCIC, B. & SKARSGARD, L. (1976) Radiosensitization and toxic effects of the 2-nitroimidazole Ro-07-0582 in hypoxic mammalian cells. *Radiat. Res.*, **67**, 459.

OZOLS, R. F., LOCKER, G. Y., DOROSHOW, J. H., GROTZINGER, K. R., MYERS, C. E. & YOUNG, R. C. (1979) Pharmacokinetics of Adriamycin and tissue penetration in murine ovarian cancer. *Cancer Res.*, **39**, 3209.

PEDERSEN, J. E., SMITH, M. R., BUGDEN, R. D. & PECKHAM, M. J. (1979) Distribution and tumour cytotoxicity of the radiosensitizer misonidazole (Ro-07-0582) in C57 mice. *Br. J. Cancer*, **39**, 429.

SIEMANN, D. W., BRONSKILL, M. J., HILL, R. P. & BUSH, R. S. (1975) The relationship between mouse arterial partial pressure of oxygen ($PaO_2$) and the effectiveness of localized tumour irradiation. *Br. J. Radiol.*, **48**, 662.

SRIDHAR, R., KOCH, C. & SUTHERLAND, R. M. (1976) Cytoxicity of two nitroimidazole radiosensitizers in an *in vitro* tumour model. *Int. J. Radiat. Oncol. Biol. Phys.*, **1**, 1149.

STRATFORD, I. J. & ADAMS, G. E. (1977) Effect of hyperthermia on differential cytotoxicity of a hypoxic cell radiosensitizer, Ro-07-0582, on mammalian cells *in vitro*. *Br. J. Cancer*, **35**, 307.

STRATFORD, I. J. & ADAMS, G. E. (1978) The toxicity of the radiosensitizer Misonidazole towards hypoxic cells *in vitro*: A model for mouse and man. *Br. J. Radiol.*, **51**, 745.

SUTHERLAND, R. M., EDDY, H. A., BAREHAM, B., REICH, K. & VAN ANTWERP, D. (1979) Resistance to Adriamycin in multicellular spheroids. *Int. J. Rad. Oncol. Biol. Phys.*, **5**, 1225.

TANNOCK, I. F. (1968) The relation between cell proliferation and the vascular system in a transplanted mouse mammary tumour. *Br. J. Cancer*, **22**, 258.

TANNOCK, I. F. (1970) Population kinetics of carcinoma cells, capillary endothelial cells, and fibroblasts in a transplanted mouse mammary tumour. *Cancer Res.*, **30**, 2470.

TANNOCK, I. F. (1978) Cell kinetics and chemotherapy: A critical review. *Cancer Treat. Rep.*, **62**, 1117.

TAYLOR, Y. C. & RAUTH, A. M. (1978) Differences in the toxicity and metabolism of the 2-nitroimidazole misonidazole (Ro-07-0582) in HeLa and Chinese hamster ovary cells. *Cancer Res.*, **38**, 2745.

THOMSON, J. E. & RAUTH, A. M. (1974) An *in vitro* assay to measure the viability of KHT tumour cells not previously exposed to culture conditions. *Radiat. Res.*, **58**, 262.

WORKMAN, P., LITTLE, C. J., MARTEN, T. R. & 4 others (1978) Estimation of the hypoxic cell sensitizer misonidazole and its O-demethylated metabolite in biological materials by reversed phase high performance liquid chromatography. *J. Chromatogr.*, **145**, 507.

YAMAURA, H. & MATSUZAWA, T. (1979) Tumour regrowth after irradiation. An experimental approach. *Int. J. Rad. Biol.*, **35**, 201.


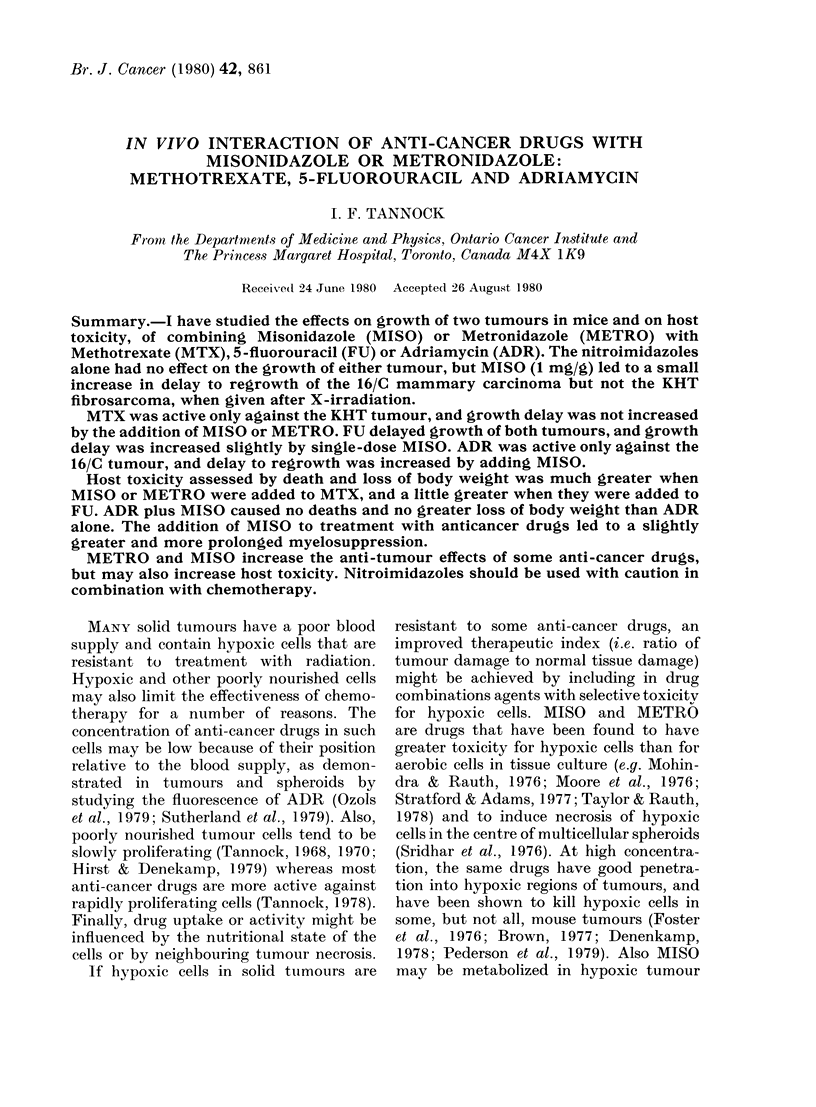

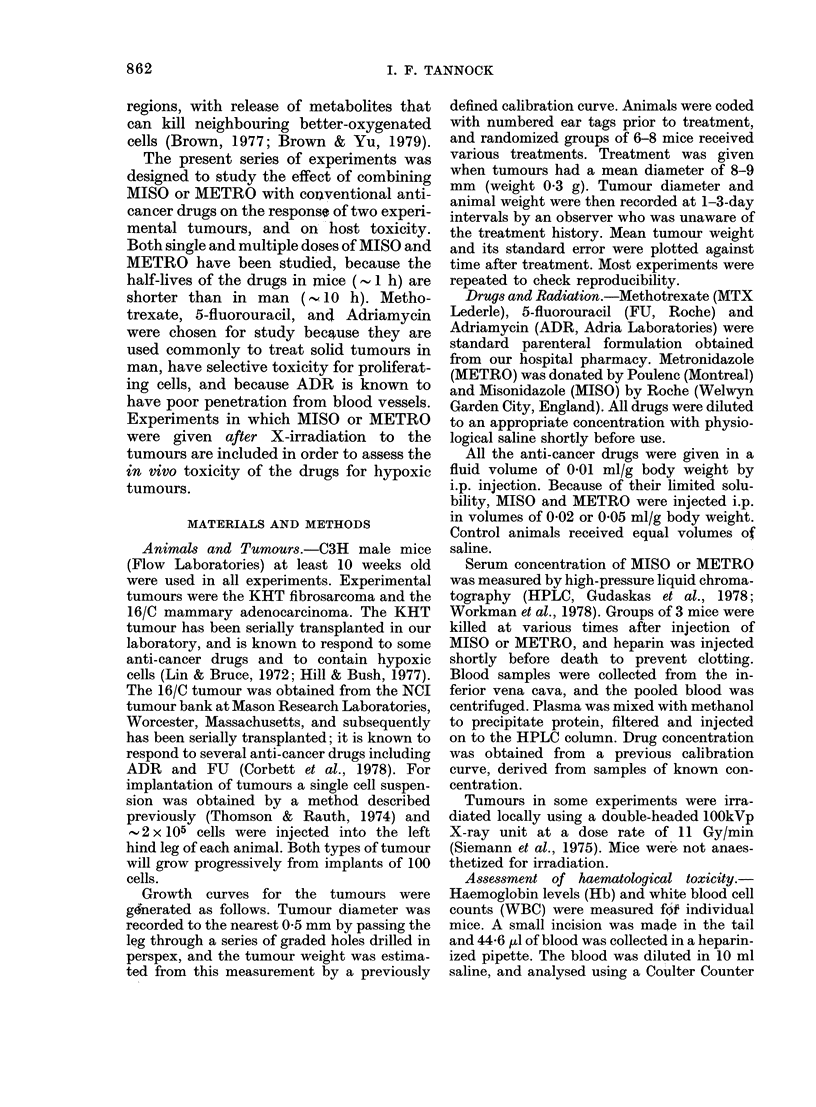

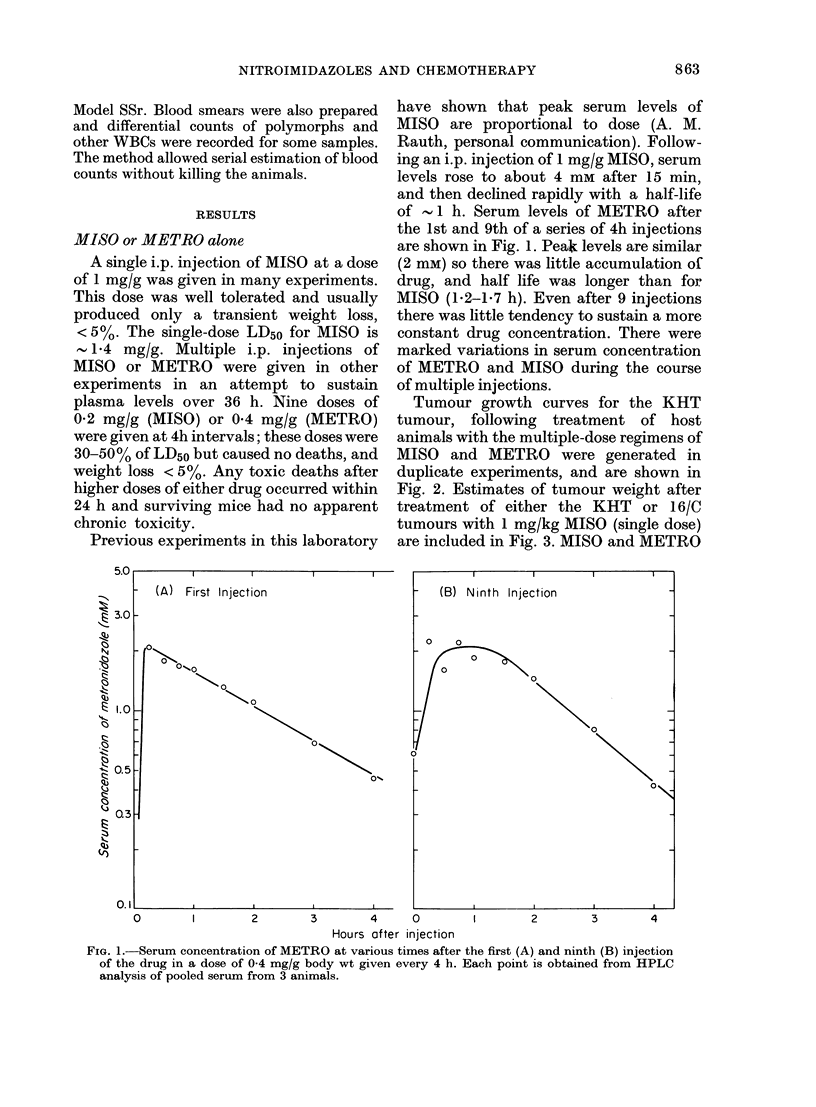

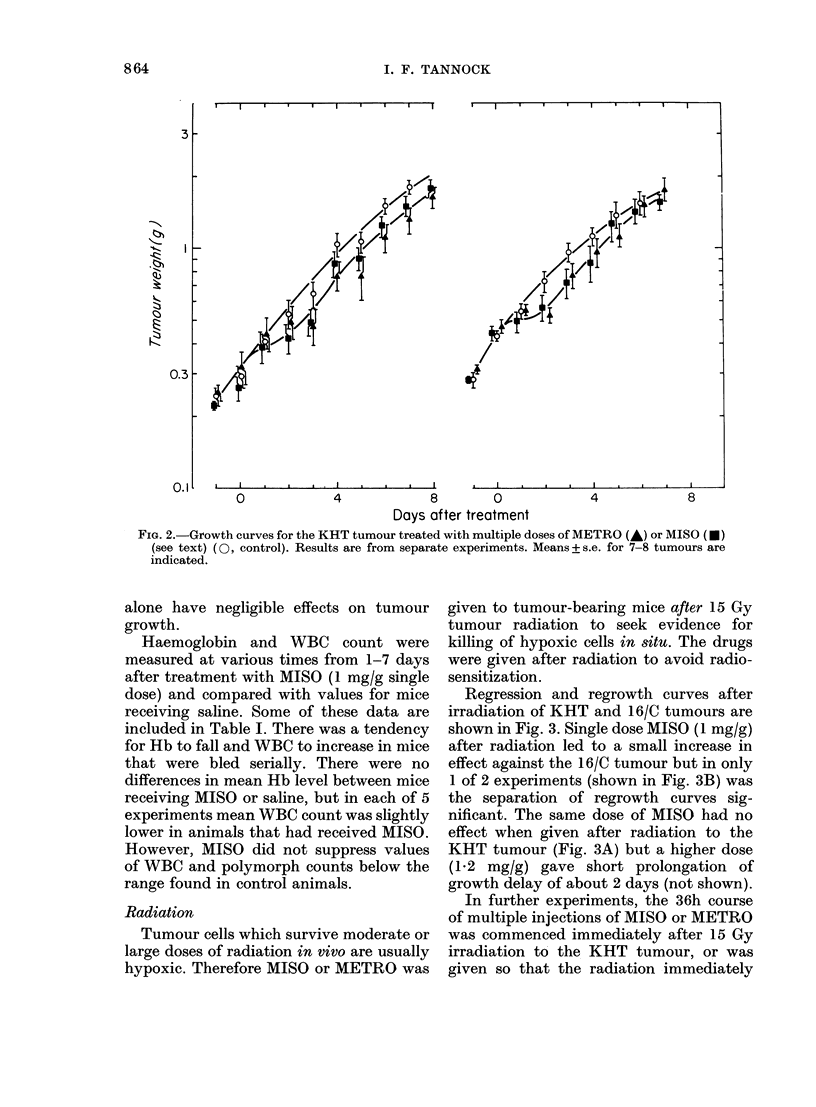

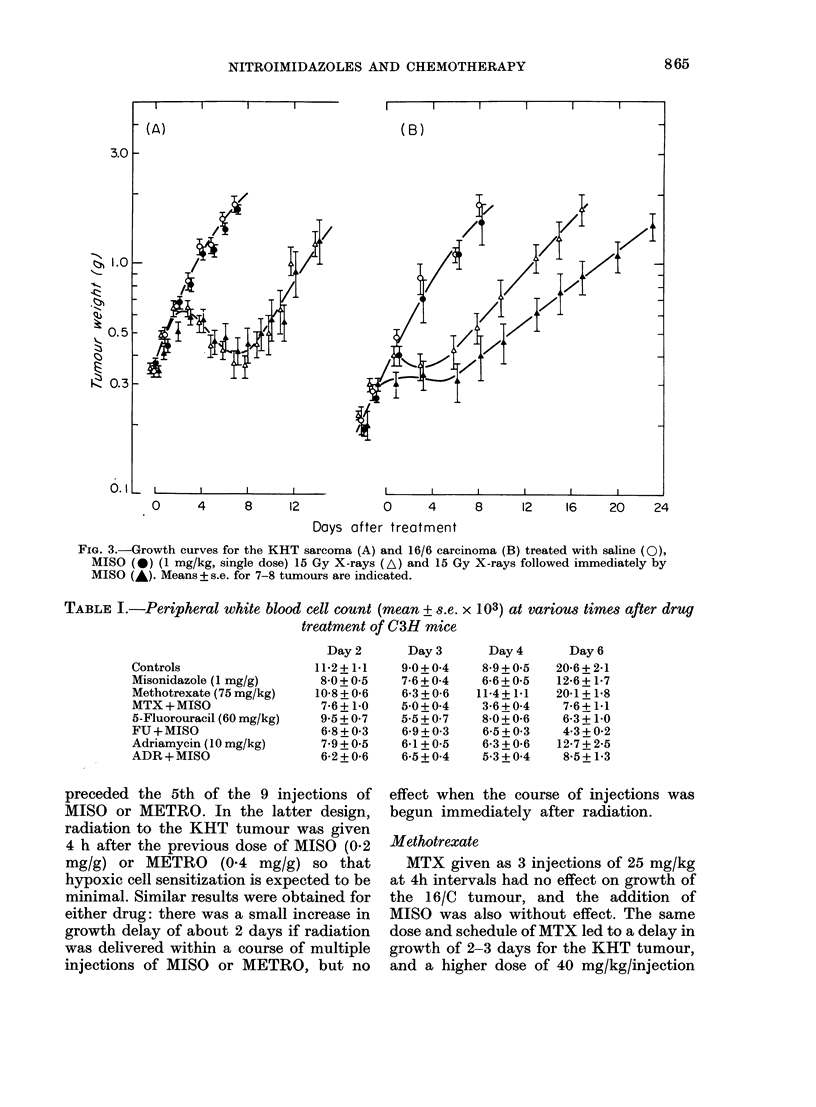

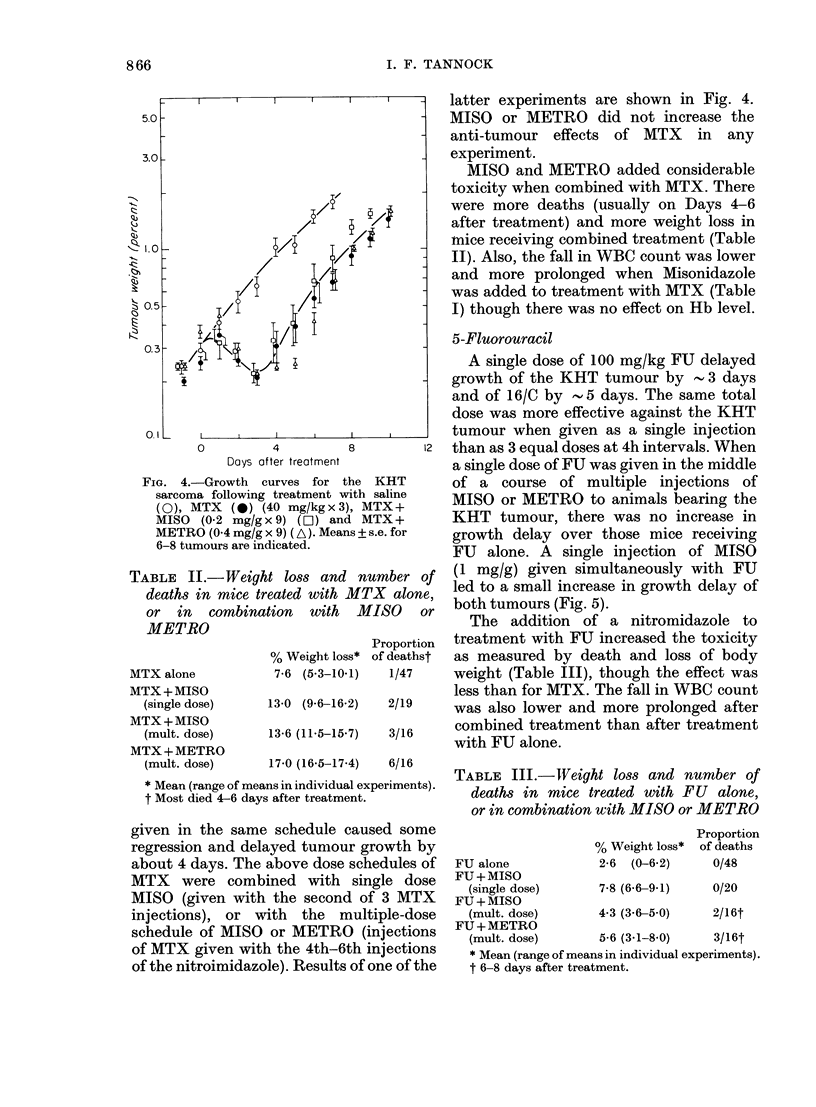

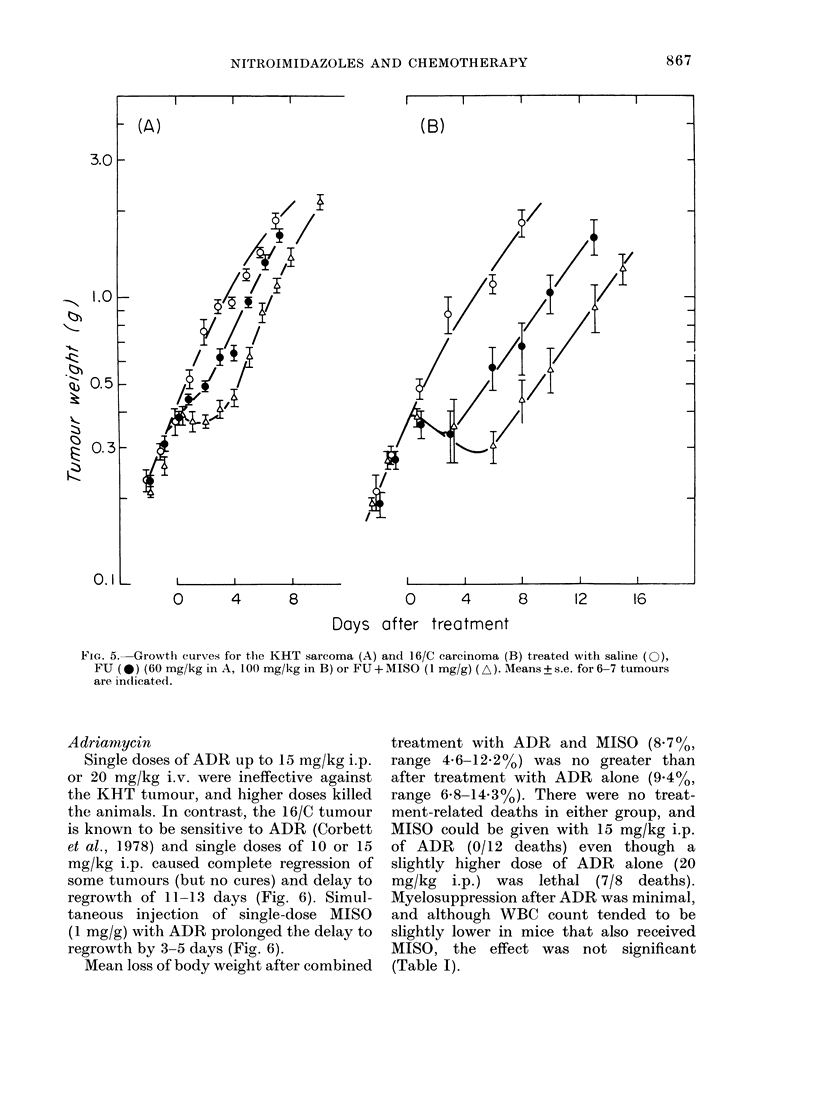

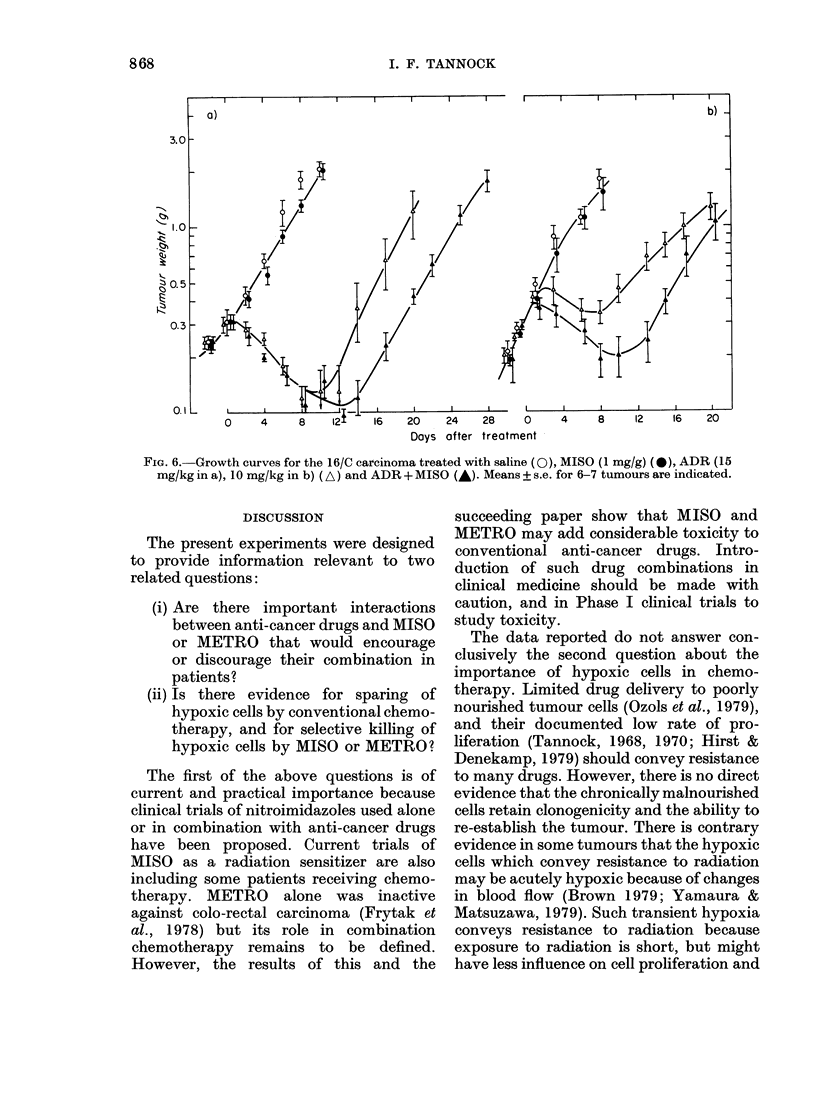

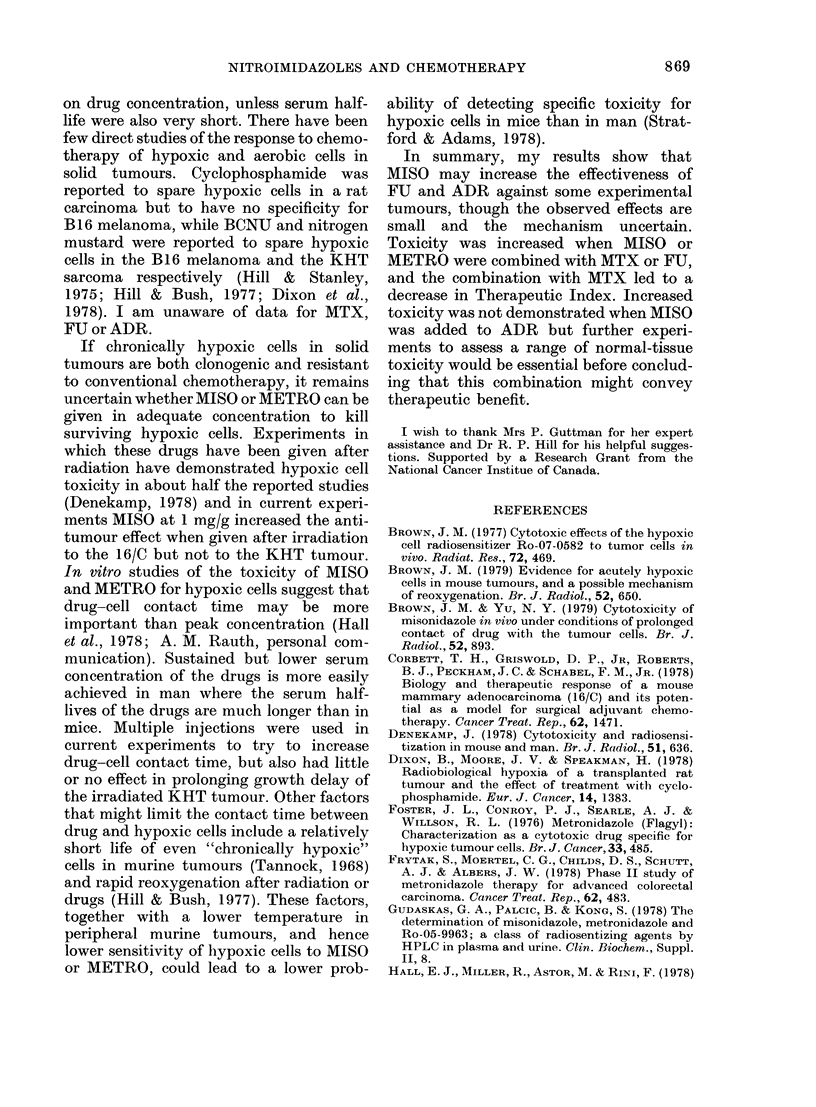

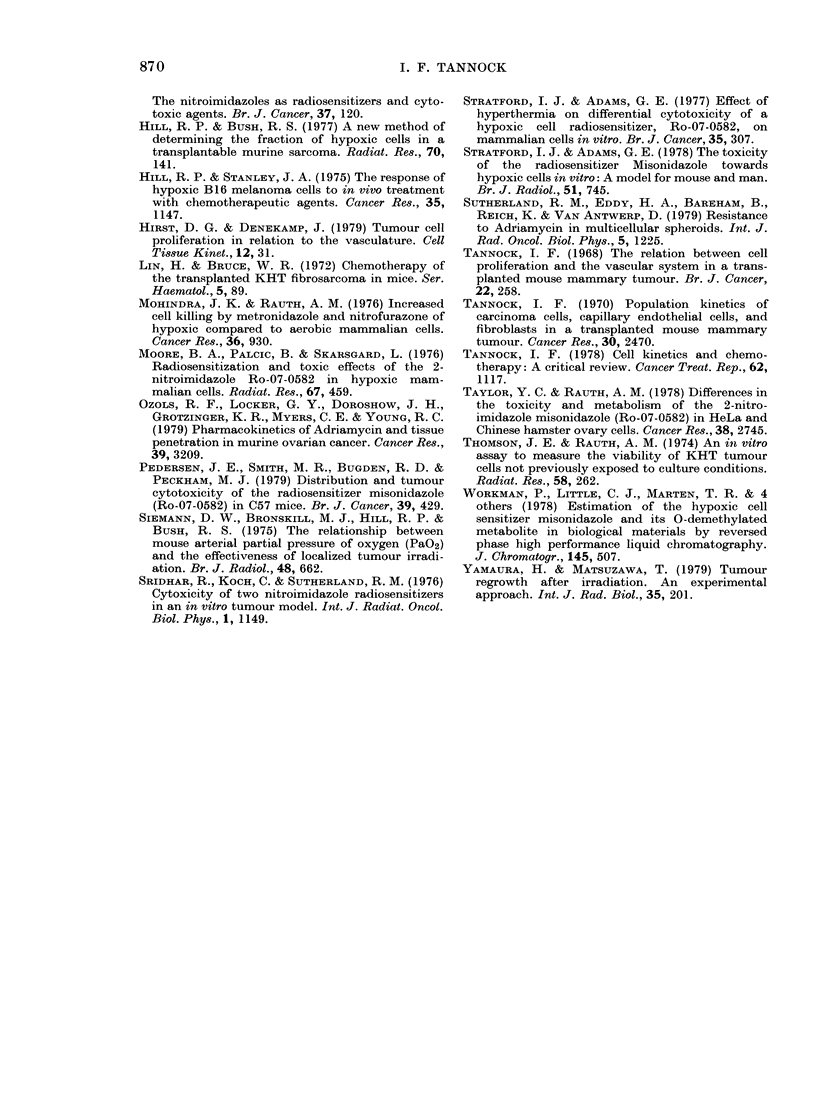


## References

[OCR_00857] Brown J. M. (1977). Cytotoxic effects of the hypoxic cell radiosensitizer Ro 7-0582 to tumor cells in vivo.. Radiat Res.

[OCR_00862] Brown J. M. (1979). Evidence for acutely hypoxic cells in mouse tumours, and a possible mechanism of reoxygenation.. Br J Radiol.

[OCR_00867] Brown J. M., Yu N. Y. (1979). Cytotoxicity of misonidazole in vivo under conditions of prolonged contact of drug with the tumour cells.. Br J Radiol.

[OCR_00873] Corbett T. H., Griswold D. P., Roberts B. J., Peckham J. C., Schabel F. M. (1978). Biology and therapeutic response of a mouse mammary adenocarcinoma (16/C) and its potential as a model for surgical adjuvant chemotherapy.. Cancer Treat Rep.

[OCR_00881] Denekamp J. (1978). Cytotoxicity and radiosensitization in mouse and man.. Br J Radiol.

[OCR_00884] Dixon B., Moore J. V., Speakman H. (1978). Radiobiological hypoxia of a transplanted rat tumour and the effect of treatment with cyclophosphamide.. Eur J Cancer.

[OCR_00890] Foster J. L., Conroy P. J., Searle A. J., Willson R. L. (1976). Metronidazole (Flagyl): characterization as a cytotoxic drug specific for hypoxic tumour cells.. Br J Cancer.

[OCR_00896] Frytak S., Moertel C. G., Childs D. S., Schutt A. J., Albers J. W. (1978). Phase II study of metronidazole therapy for advanced colorectal carcinoma.. Cancer Treat Rep.

[OCR_00909] Hall E. J., Miller R., Astro M., Rini F. (1978). The nitroimidazoles as radiosensitizers and cytotoxic agents.. Br J Cancer Suppl.

[OCR_00916] Hill R. P., Bush R. S. (1977). A new method of determining the fraction of hypoxic cells in a transplantable murine sarcoma.. Radiat Res.

[OCR_00922] Hill R. P., Stanley J. A. (1975). The response of hypoxic B16 melanoma cells to in vivo treatment with chemotherapeutic agents.. Cancer Res.

[OCR_00933] Lin H., Bruce W. R. (1972). Chemotherapy of the transplanted KHT fibrosarcoma in mice.. Ser Haematol.

[OCR_00938] Mohindra J. K., Rauth A. M. (1976). Increased cell killing by metronidazole and nitrofurazone of hypoxic compared to aerobic mammalian cells.. Cancer Res.

[OCR_00944] Moore B. A., Palcic B., Skarsgard L. D. (1976). Radiosensitizing and toxic effects on the 2-nitroimidazole Ro-07-0582 in hypoxic mammation cells.. Radiat Res.

[OCR_00950] Ozols R. F., Locker G. Y., Doroshow J. H., Grotzinger K. R., Myers C. E., Young R. C. (1979). Pharmacokinetics of adriamycin and tissue penetration in murine ovarian cancer.. Cancer Res.

[OCR_00957] Pedersen J. E., Smith M. R., Bugden R. D., Peckham M. J. (1979). Distribution and tumour cytotoxicity of the radiosensitizer misonidazole (Ro-07-0582) in C57 mice.. Br J Cancer.

[OCR_00962] Siemann D. W., Bronskill M. J., Hill R. P., Bush R. S. (1975). The relationship between mouse arterial partial pressure of oxygen (PaO2) and the effectiveness of localized tumour irradiation.. Br J Radiol.

[OCR_00969] Sridhar R., Koch C., Suterland R. (1976). Cytotoxicity of two nitroimidazole radiosensitizers in an in vitro tumor model.. Int J Radiat Oncol Biol Phys.

[OCR_00975] Stratford I. J., Adams G. E. (1977). Effect of hyperthermia on differential cytotoxicity of a hypoxic cell radiosensitizer, Ro-07-0582, on mammalian cells in vitro.. Br J Cancer.

[OCR_00981] Stratford I. J., Adams G. E. (1978). The toxicity of the radiosensitizer misonidazole towards hypoxic cells in vitro: a model for mouse and man.. Br J Radiol.

[OCR_00987] Sutherland R. M., Eddy H. A., Bareham B., Reich K., Vanantwerp D. (1979). Resistance to adriamycin in multicellular spheroids.. Int J Radiat Oncol Biol Phys.

[OCR_00999] Tannock I. F. (1970). Population kinetics of carcinoma cells, capillary endothelial cells, and fibroblasts in a transplanted mouse mammary tumor.. Cancer Res.

[OCR_00993] Tannock I. F. (1968). The relation between cell proliferation and the vascular system in a transplanted mouse mammary tumour.. Br J Cancer.

[OCR_01005] Tannock I. (1978). Cell kinetics and chemotherapy: a critical review.. Cancer Treat Rep.

[OCR_01010] Taylor Y. C., Rauth A. M. (1978). Differences in the toxicity and metabolism of the 2-nitroimidazole misonidazole (Ro-07-0582) in HeLa and Chinese hamster ovary cells.. Cancer Res.

[OCR_01015] Thomson J. E., Rauth A. M. (1974). An in vitro assay to measure the viability of KHT tumor cells not previously exposed to culture conditions.. Radiat Res.

[OCR_01021] Workman P., Little C. J., Marten T. R., Dale A. D., Ruane R. J., Flockhart I. R., Bleehen N. M. (1978). Estimation of the hypoxic cell-sensitiser misonidazole and its O-demethylated metabolite in biological materials by reversed-phase high-performance liquid chromatography.. J Chromatogr.

[OCR_01029] Yamaura H., Matsuzawa T. (1979). Tumor regrowth after irradiation; an experimental approach.. Int J Radiat Biol Relat Stud Phys Chem Med.

